# A New Classification of Perceptual Interactions between Odorants to Interpret Complex Aroma Systems. Application to Model Wine Aroma

**DOI:** 10.3390/foods10071627

**Published:** 2021-07-14

**Authors:** Vicente Ferreira, Arancha de-la-Fuente-Blanco, María-Pilar Sáenz-Navajas

**Affiliations:** 1 Laboratorio de Análisis del Aroma y Enología (LAAE), Department of Analytical Chemistry, Instituto Agroalimentario de Aragón (IA2) (UNIZAR-CITA), Universidad de Zaragoza, Associated Unit to Instituto de las Ciencias de la Vid y del Vino (ICVV) (UR-CSIC-GR), c/Pedro Cerbuna 12, 50009 Zaragoza, Spain; arandlfb@unizar.es; 2 Department of Enology, Instituto de Ciencias de la Vid y del Vino (CSIC-GR-UR), Finca La Grajera, E-26007 Logroño, La Rioja, Spain; mpsaenz@icvv.es

**Keywords:** odours, aroma vectors, sensory perception, odorants, quality, interaction, suppression, enhancement, modelling, perceptual integration

## Abstract

Although perceptual interactions are usually mentioned and blamed for the difficulties in understanding the relationship between odorant composition and aromatic sensory properties, they are poorly defined and categorised. Furthermore, old classifications refer mainly to effects on the odour intensity of the mixture of dissimilar non-blending odours and do not consider odour blending, which is one of the most relevant and influential perceptual interactions. Beginning with the results from classical studies about odour interaction, a new and simple systematic is proposed in which odour interactions are classified into four categories: competitive, cooperative, destructive and creative. The first categories are most frequent and display a mild level of interaction, being characterised mostly by analytical processing. The last two are less frequent and activate (or deactivate) configurational processes of object recognition with deep effects on the quality and intensity of the perception. These interactions can be systematically applied to interpret the formation of sensory descriptors from the odorant composition, suggesting that qualitatively the system works. However, there is a lack of quantitative data to work with odour intensities reliably, and a pressing need to systematise the effects of creative interactions.

## 1. Introduction

Odours, involved in both orthonasal smell and retronasal aroma, influence our affective state (i.e., mood and emotions), well-being and behaviour in everyday situations [1,2,3], including dietary behaviours. Odours are largely involved in flavour formation, modulate preference and liking [4] defining our patterns of food and beverage consumption. Odour is relevant in shaping food perception by modulating physiological phenomena linked to nutrient–flavour associations [5]. Not anecdotally, the sense of smell has the greatest discriminative capacity among the senses [6] and conveys a large and significant part of the qualitative information associated to the perceived qualities of food and thus to its perceptual identity [7], consistently with the biological functions of smell [8,9]. This discriminative capacity requires a highly complex detection system, which in humans is constituted not only by the most complex and diverse receptor network [10], but also by several layers of signal modulation (olfactory bulb) [11] and by a highly complex signal re-interpretation system (brain) [12] in close connexion with the cerebral reward system [4,13].

However, the relevance of the sense of smell is underestimated and little considered in daily life. On the one hand, most people still believe that humans have a poor sense of smell in terms of sensory capabilities, in comparison with other mammals such as dogs or rodents. However, this fact has been proved to be a myth [14]. On the other hand, encoding odours and flavours is highly influenced by cognitive processes [15], including learning. Odour knowledge can be acquired by implicit or perceptual learning (i.e., mere exposure to odours) and more importantly by associative learning (i.e., association of odours to taste, visual or verbal labels) [16]. Learning to name odours influences odour perception and brain activation [17]. However, it is a more demanding task than naming objects, for example, and thus requires specific training for achieving discriminant and stable flavour perception, i.e., olfactory expertise [16]. In fact, only technical experts belonging to specific domains, such as perfumers, flavourists, oenologists, cooks or sommeliers have considered olfaction as an essential tool and have developed awareness, vocabulary and specific cognitive strategies to deal with this sense. In recent years, a small fraction of the population of the most developed countries, generally belonging to the cultural elite [18], have regained consciousness of the importance and benefits associated to training the sense of smell to enjoy what, not in vain, is known as “culinary arts”.

In spite of its relevance, the understanding of the process of the integration of the signals elicited by the odorants reaching our nostrils, well during orthonasal olfaction or via retronasal during food intake, to form different odour notes perceived is nowadays quite limited. It is widely accepted that such integration involves two main cognitive processes: bottom-up (i.e., obtain information of the characteristics of the product at the surface level: “smelling before thinking”) and top-down (interpret the olfactory signal based on previous experience: “thinking before smelling”) being both interconnected and modulated by the characteristics of the individual and the stimulus [15,19,20,21]. This review focuses on the understanding of the integration of olfactory signals at stimulus level, and will try to show how the rationalisation and categorisation of perceptual interactions provide stable rules able to explain the odour signals from the odorant composition.

Surprisingly and to the best of our knowledge, there is not a clear and unequivocal definition of what is understood as perceptual “interactions” between odours, or about the different types of phenomena included within the concept. Moreover, the classifications derived from the old psychophysical studies (hyper-addition or synergy, complete addition, partial addition, compromise and subtraction) refer exclusively to the effects on the intensity of the mixture of dissimilar and non-blending odours and do not consider odour blending, which represents the most relevant level of perceptual interaction. In odour blending, complex stimuli, originally constituted by several heterogeneous percepts, is converted into a homogeneous percept. As it will be shown, the outcome can be the perception of a new odour, or the perfection of an already present one, making it closer to a known odour object stored in our memory. In both cases, the interaction has a profound impact on the qualitative and quantitative properties of the odours perceived in the mixture.

For dealing with blending effects, we should rely on those odour concepts stored in our memory and make use of some elements derived from image recognition which help understanding how the human brain processes and integrates information to recognise known objects in a complex image. This makes it possible to propose a new and simple classification of perceptual interactions including four categories: competitive, cooperative, destructive and creative. It will be shown that the classification is able to accommodate all previous results from classical psychophysics, and its potential to explain the aroma of complex products will be preliminary illustrated by its application to the interpretation of the aroma of the wine.

## 2. Essential Concepts about Olfaction, Odours and Odorants

### 2.1. Definition of Odorants, Odours, Odour Objects and Odour Concepts

One of the most repeated clichés in the study of odours is that most of them are not made up of a single odorant but are mixtures of several of them [22]. At the chemical receptor level, there will be quite a few obvious differences between the monomolecular odour of synthetic vanilla, for example, and the complex odour associated with curry, formed by a complex mixture of many different odorants. However, it seems that the brain does not care much about this difference, and in both cases, odours will be identified and further processed as single entities. The integration of a complex mixture of odorants into a single odour will take place, regardless of its chemical complexity, insofar as it represents a familiar object or product [21]. For example, the aroma of many fruits is composed of several dozens of odorants displaying quite different odours, yet the specific odour of such fruit is identified as a single concept. This integration ability must be kept in mind for addressing the odour properties of mixtures of odorants. 

At this point, it is convenient to set some basic definitions:

Odorant: In general, it is a molecule with odour. In a specific product, it is any volatile molecule present at a concentration not much below its odour threshold. Arbitrarily, we can suggest, Ci > 0.1 × Ti, i.e., any volatile present in a product (i) at a concentration (Ci) above 0.1 its threshold (Ti) will be considered an odorant in such a product. 

Odour: Perception associated with the detection of one or more odorants associated or not with known odour objects. In the former case, the odour is recognised and perceived synthetically, in the latter case will be perceived analytically.

Odour object: The object or product that emits or is associated with an odour.

Odour concept -or olfactory concept: The set of information stored in our brain associated to a specific and familiar odour object.

Basic attributes or “features”: A set of relatively generic descriptors (sweet, fruity, floral, fresh, green, sharp, fishy, musty, mouldy, chemical, burned, toasted, lactic...) identified in the odour object often used in the analytical definition of the different unidentified odours. These basic attributes or “features” could be related to primary odours [23], a controversial concept used in the past [24]. 

### 2.2. About Odour Concepts and Their Identification

Many odour concepts are experienced and stored in memory during earliest childhood before the development of language (i.e., first odour–episode associations), which may explain why many of them are linked to olfactory representations not associated with specific semantic labels. This fact could explain why there is a generalised difficulty in verbalising odour perception. In addition, odour concepts are acquired in context with all the concurring contextual information (situation, visual cues, texture, touch, taste...) yielding to inseparable learnt associations, which explain the difficulty in the identification of familiar odours when the other contextual information is lost. A consequence of this associative learning [16] is that odour concepts, in the absence of specific training and flavour-friendly familiar backgrounds, can be blurred and poorly refined, which adds difficulties both in recognition and verbal description [16,25].

It can then be hypothesised that owning a well-defined odour concept, i.e., olfactory mental representation, is a prerequisite for being able to identify its associated odour object in a complex mixture. Such identification implies the segregation of the different elements in the mixture into the figure (the identified odour object) and the background (the rest of odorants in the mixture). Both figure and background are part of the so-called perceptual image. Figure refers to a solid and structured object that captures main attention, i.e., focal image as defined by Valentin [26], while background is the surrounding that remains blurred “behind” the focal image. The comparison between odour objects, in odour and flavour perception, and figures, in the interpretation of visual images, can be further extended to incorporate some key elements of image processing.

1. In the cases in which two different figures can be identified in a same image, the attention can shift from one to the other quickly and continuously. Therefore, even if the perceptual process acts in two different phases; one focussing on one of the figures and segregating the second one into the background, and a second opposite one in which the second figure is brought into focus and the first segregated; the two objects are identified and perceived as being together.
2. Often the figure is composed of sets of self-standing elements which will be grouped into a unified whole [26]. This unified whole can be perceived even if its associated figure is not perfect or if it is incomplete [27].
3. The perception can be either analytical or configurational. In “analytical mode” the attention is directed towards the single features composing the different figures in the image [28], while in “configurational mode” the attention is directed towards the figures in the image.

## 3. The Odour Properties of Binary Mixtures of Odours

For reaching a reasonable definition and categorisation of the different perceptual interactions, we should first review old psychophysical works showing the different outcomes of mixing odorants, observing which ones are the most frequent and which are rare, as reviewed by Ferreira [29,30].

### 3.1. Binary Mixtures Are Governed by Intensity Ratios

The rules governing the outcome of the binary mixtures of two dissimilar non-blending odours derive from the studies carried out by Laing et al. [31] and further refined by Olsson [32], Cain et al. [33] and by Atanasova et al. [34] and summarised by Ferreira [29]. A key idea to deal with mixtures of odours is to work with intensities, as these are the real psychophysical parameters, and not with concentrations, normalised or not by thresholds. A further key premise is that mixtures of two odorants A and B at different intensities but similar intensity ratios $I_{A}$/$I_{B}$ have the same odour qualities. This premise has been experimentally validated [31,32,35] and finds also support in the everyday finding that it is possible to recognise odours composed of mixtures of odorants at different levels of concentrations, as it happens when the scents emitted by flowers, plants or by the cooking pot in the kitchen are smelled at different distances from the source [36].

The composition of the mixture AB will be expressed in odour terms by its two odour fractions, $\tau_{A}$ and $\tau_{B}$, which, as defined in Equations (1) and (2), represent the relative amounts of each odour present in the mixture:

(1) $$\tau_{A}=\frac{I_{A}}{I_{A}+I_{B}}$$

(2) $$\tau_{B}=\frac{I_{B}}{I_{A}+I_{B}}$$

The total intensity of the mixture is $I_{AB}$. This intensity, normalised by the sum of the intensities of the individual components in isolation, is usually denoted as σ, and represents the fraction of the odour intensity surviving in the mixture (Equation (3)).

(3) $$\sigma =\frac{I_{AB}}{I_{A}+I_{B}}$$

This parameter can then be represented versus the odour fractions $\tau_{A}$ or $\tau_{B}$ as suggested by Cain and Drexler [37] and Patte and Laffort [38], as will be seen in the next section.

Regarding the quality of the odour of a binary mixture of two dissimilar non-blending odours, *A* and *B*, the mixture will smell either mainly as *A* or *B*. The odour of this mixture can then be easily represented by measuring the perceived intensities of *A* and *B* in the mixture. Let us denote them as $I_{A/AB}\;$ or $I_{B/AB}$. These intensities can be expressed as the fraction of odour *A* perceived in the mixture ($\tau {{}^{\prime }}_{A})$ or as its complementary, the fraction of odour *B* perceived in the mixture $\left(\tau {{}^{\prime }}_{B}=1-\tau {{}^{\prime }}_{A}\right)$, simply by dividing them by the summation of both odour intensities, as indicated in Equations (4) and (5):

(4) $$\tau {{}^{\prime }}_{A}=\frac{I_{A/AB}}{I_{A/AB}+I_{B/AB}}$$

(5) $$\tau {{}^{\prime }}_{B}=\frac{I_{B/AB}}{I_{B/AB}+I_{A/AB}}$$

These odour fractions can now be represented versus the corresponding compositional odour fractions $\tau_{A}$ or $\tau_{B}$, as first proposed by Cain, Schiet, Olsson and deWijk [33]. Some authors have obtained nearly completely equivalent representations measuring the frequency with which the odour of the mixtures was identified by the panellists as *A*, as *B* or as both simultaneously [32].

### 3.2. The Odour Intensity of a Binary Mixture

**Figure 1:**
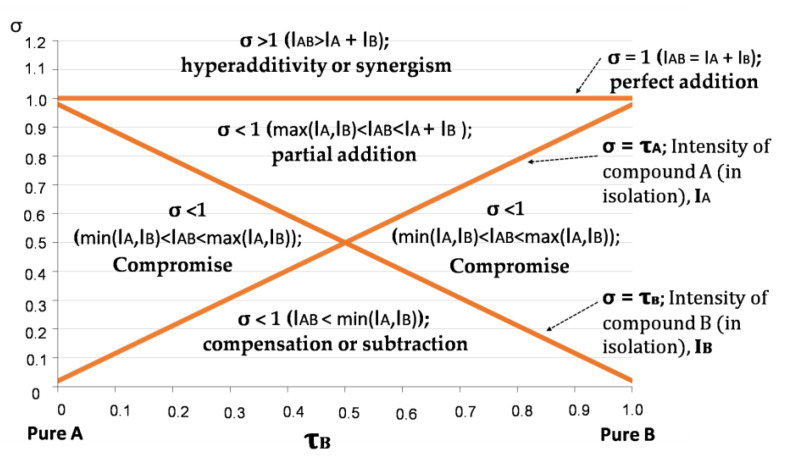
Plot with the representation of σ vs. τ with the most probable results according to the intensity values in the binary mixture, as proposed by Cain and Drexler [37]. Adapted with permission from ref. [30].

The normalised global odour intensities of the binary mixtures, σ, are represented in the σ  − τ plot given in Figure 1. These plots have three master lines. The horizontal one corresponds to an ideal situation in which σ = 1 at any odour composition, i.e., the measured odour of the mixture, $I_{AB}$ is always the sum of the intensities of the two components present ($I_{AB}$ =$\;I_{A}$ + $I_{B}$). The diagonal lines correspond to the cases in which $I_{AB}$ = $I_{A}$ or $I_{AB}$ = $I_{B}$, respectively. The areas delimited by these lines are:

1. the hyper-addition or synergism zone, in which the intensity of the mixture is higher than the sum of the intensities of the isolated compounds ($I_{AB}$ > $\;I_{A}$ + $I_{B}$; σ > 1);
2. the partial addition area, in which the intensity of the mixture is higher than the intensity of the most intense component, but smaller than the sum of the intensities of the two components;
3. two compromise areas, where compromise means reduction. In these areas, the intensity of the mixture is smaller than the intensity of the most intense component, but higher than the intensity of the least intense;
4. the compensation or subtraction area, in which the intensity of the mixture is smaller than the intensity of the least intense component.

**Figure 2:**
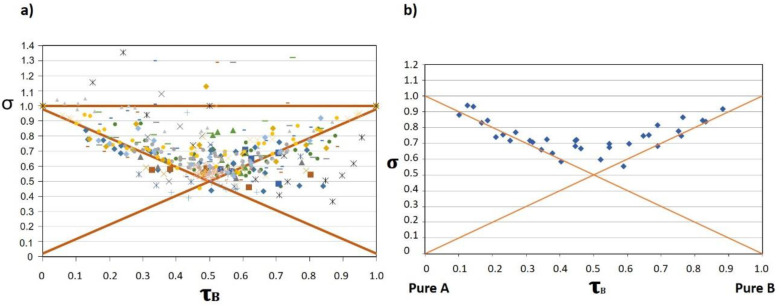
Representation of 520 different mixtures derived from 198 pairs of AB odorants reported in the scientific literature and compiled by Ferreira [30]. (**a**) General σ vs. τ plot showing the probability of occurrence of the different interactions; (**b**) most probable representation for a σ vs. $\tau_{B}$ according to a real mixture pyridine/butanol proposed by Olsson [32]. Adapted with permission from ref. [30].

The 520 different mixtures derived from 198 pairs of AB odorants reported in the scientific literature and compiled by Ferreira [30] are represented together in a single σ−τ plot (Figure 2a). The analysis of this relatively large set of experimental data makes it possible to draw the following conclusions about the relative frequencies with which the different outcomes occur:

1. The most frequent case is that of partial addition, which takes place in more than 70% of the mixtures.
2. Compromise is not dominant, but it is a relatively common outcome, since around 25% of the mixtures represented in the plot are in the compromise area. It should be noted that in the AB mixtures in which compromise is observed, it affects only to one of the components; i.e., compromise is observed at low $\tau_{B}$ or at low $\tau_{A}$, but not at both sides. This implies that compromise is found in around 12.5% of the cases.
3. Hyperaddition is rare. Only 8 out of the 520 cases (1.5%) represented in the plot. All but one of these cases correspond to mixtures of odorants at very low intensities, close to the threshold area. Hyperaddition is a likely outcome only at very low intensity levels. The rare case of hyperaddition at supra-threshold area can be assigned to a strong perceptual interaction.
4. Subtraction has not been so far reported. The three dots in this area correspond to solutions at τ close to 0.5 for which σ was not significantly smaller than that of the compromise area.

Figure 2b corresponds to a real example built with the pyridine/butanol pair examined by Olsson [32]. This case can be considered to be representative of the most common and quasi-ideal and perfectly symmetric case. As can be seen, the addition of the B component on solutions dominated by A ($I_{A}$ > $I_{B}$, $\tau_{B}$ < 0.4), or the addition of A on solutions dominated by B ($I_{B}$ > $I_{A}$, $\tau_{B}$ > 0.6), has very little effect on the intensity of the mixture, i.e., the addition of a second less intense odour to any odorous solution has as most common effect on the intensity… none!. This effect was first recognised by Laffort and Dravnieks [39] who named it as “the strongest component model”. When the intensities of the two odorants mixed are similar (0.4 < $\tau_{A}$, $\tau_{B}$ < 0.6), the intensity of the mixture is higher than the intensity of the most intense component, but the increase is, on average, just a 19%, equivalent to the effect associated to doubling the concentration of an odorant with Stevens exponent of just 0.22, while normal odorants have exponents between 0.3 and 0.6 [29]. The addition of a third or fourth additional isointense odorant to the mixture has no further effect on the intensity as has been demonstrated in different reports [39,40,41,42,43], which suggests that different non-blending odours (i.e., heterogeneous percepts) are not additive.

The less frequent cases of compromise and hyperaddition will be treated in the section dealing with perceptual interactions.

### 3.3. The Odour Quality of a Binary Mixture

**Figure 3:**
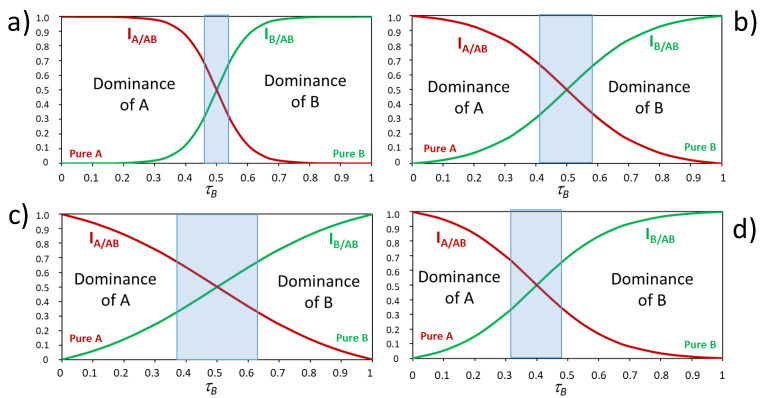
Idealised representations relating the intensity of the perceived odours in a binary mixture of non-blending odorants to its odour composition. The plots a–c represent the steepest ((**a**), upper left), mean ((**b**), upper right) and the flattest ((**c**), left down) sigmoid functions found experimentally with a perfectly symmetric dominance, respectively. The right-down plot (**d**) represents the strongest asymmetry reported. Adapted with permission from ref. [29].

The quality of the odours of binary mixtures of non-blending odours is represented in the plots $\tau {{}^{\prime }}_{A}\;\;\tau {{}^{\prime }}_{B}\;$ vs. $\tau_{B}$ as those shown in Figure 3.

The four plots represent four different possible idealised but real outcomes, differing in the sharpness of the transition from perceiving one component to perceiving the other (Figure 3a–c) or in the symmetry (Figure 3d). Experimental results demonstrate that the odour of a binary mixture is essentially well represented by the odour of its most intense component, except in the areas of co-dominance in which both odours can be perceived. Sharp transitions (Figure 3a) from one of the odours to the second with very small co-dominance areas are generally observed when the odours mixed are very similar and in one case of demonstrated antagonism at receptor level [44,45,46]. Smooth transitions (Figure 3c) are more typical of very dissimilar odours in which co-dominance areas are largest [31,46,47]. In practical terms, this means that for completely masking an odorant with a very dissimilar odour, such as pyridine with limonene, we need to add an intensity of the limonene nearly twice that of pyridine.

Regarding the transition point, earlier researchers believed that in most cases the plots were completely symmetric, such as those shown in Figure 3a–c, so that at isointensity (τ = 0.5 in the figure) both odours were equally perceived. However, it was later demonstrated that in many cases there was some asymmetry, so that at isointensity one of the odours was already more clearly perceived than the other. The extreme case shown in Figure 3d corresponds to the idealised representation of the mixture of propanoic acid (A odour) and the mint-smelling carvone (B odour) [31]. The isointense mixture of these two components is completely dominated by carvone, which accounts for more than 66% of the perceived odour, i.e., carvone masks strongly the odour of propanoic, while this one can hardly mask that of carvone.

## 4. Perceptual Interactions between Odours

The data summarised in the previous section, together with data from other reports [21,22,31,40,48] and our own experience, make it possible to define the following categories of perceptual interactions:

1. Competitive
2. Cooperative
3. Destructive
4. Creative

The previous list ranks the different categories of perceptual interactions following a descendent order of occurrence and an ascendant order regarding the intensity and complexity of the interaction. For supra-threshold dissimilar and non-blending odours, competitive interactions are the most current outcome, but for similar odours, particularly at threshold levels, cooperative interactions will be the most likely outcome. These two interactions are the mildest from the point of view of the intensity of the perceptual interaction, in the sense that their qualities can be easily explained from the original odours and their intensities derive from the mathematical properties of the corresponding psychophysical plots. These two types of interactions are related to analytical processing and are mainly governed by bottom-up perceptual strategies.

The last interactions are less frequent. Destructive interactions, which as will be shown are related to the existence of compromise in intensity, can be observed in 12% of the mixtures, while creative interactions are numerically insignificant, although they are completely essential for flavour modelling and formulation. In these two types of interactions, the changes in the intensity and/or quality of the odour associated to the mixture cannot be derived from the properties of the original odours, implying a much stronger perceptual processing related to configurational or synthetic processes mainly governed by top-down perceptual strategies. 

For the sake of homogeneity, competitive and destructive interactions will be first commented.

### 4.1. Competitive Interactions

**Figure 4:**
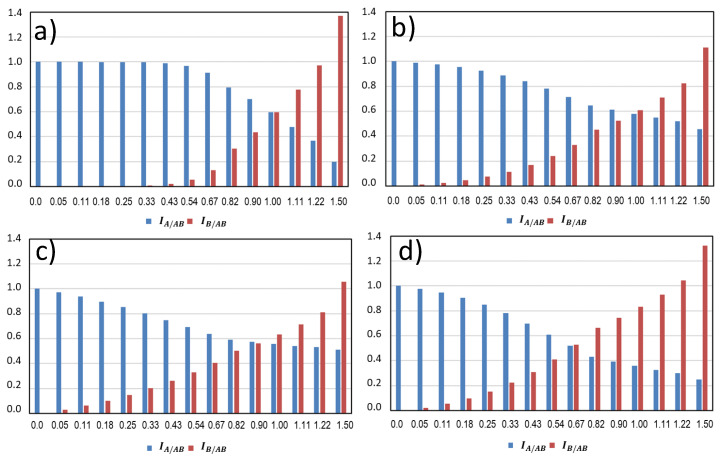
Modelled effects of the addition of increasing levels, in odour intensity units, of a second component (B—in red) on a solution containing a fixed amount of odour A—in blue ($I_{A}$ = 1). The plots represent the odour intensities of the two component odours ($I_{A/AB}$ and $I_{B/AB}$) in four situations differing in the steepness of the transition from one odour to the second ((**a**) represents the steepest curves and sharpest transition, (**b**) the mean and (**c**) the flattest curves and transitions) or on the asymmetry (**d**). In this last case, the odour added is less dominant. The additivity of intensities follows in all cases a partial addition law.

Most of the pairs of odours we can mix are dissimilar and non-blending, and their binary mixtures at suprathreshold levels follow a competitive interaction pattern, which is the natural outcome of partial addition, as shown in Figure 2b, regardless of the type of dominance found in the mixture (Figure 3). This statement is demonstrated with the representations given in Figure 4. The figure represents the perceived intensities of A and B odours in binary mixtures created by adding different amounts, in odour-intensity terms, of the B odour to a solution containing a unity of odour A. The plots have been built by assuming that the overall intensity of the mixture is $I_{AB}\;$ = σ × ($I_{A}\;$ + $I_{B}$); as follows from Equation (3), and that σ is well represented by a Stevens auto-addition function with an exponent *n* = 0.22; for the dominance, the four different types of dominance patterns given in Figure 3 are represented in the four plots.

It can be appreciated, in spite of notorious differences between the four plots, that in all cases there is a neat decrease of the odour intensity of the A component in parallel with the increase of the intensity of the B odour. Overall, it is evident that the intensity gained by B odour is lost by the A odour, which is why this pattern should be named “competitive interaction”.

It is the mildest and most common form of interaction. As already mentioned, this is the simple consequence of the inability of our brain to focus in two different focal images simultaneously [15,49]. Our attention shifts from one to the other quickly and continuously, even being the intensity related to the relative sizes of the two images.

### 4.2. Destructive Interactions

**Figure 5:**
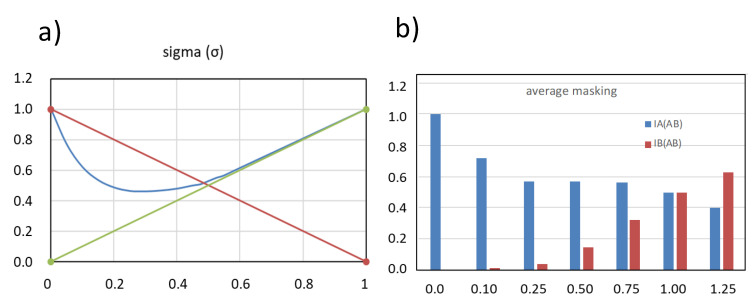
Destructive interactions. (**a**) corresponds to the σ−τ plot of a mixture of 2 odours displaying strong compromise effects (the addition of B on A has as a result a net decrease of the odour intensity. (**b**) shows the odour intensities of the two component odours ($I_{A/AB}$ and $I_{B/AB}$) in mixtures containing a fixed amount of A ($I_{A}$ = 1) and increasing amounts of B (from $I_{B}\;$= 0 to $I_{B}\;$= 1.25). For this plot a sigmoid with a = 0.30 (average masking effect) and b = 0.50 (perfect symmetry) was considered.

Destructive interactions are those in which the addition of a second odour (B) to a first one (A) causes a decrease in the perceived intensity of A without any clear increase in the intensity of the added odour, B. This is the direct and unavoidable consequence of the existence of compromise, that, as previously mentioned, occurs in 12.5% of the mixtures. This can be easily demonstrated as it was done in the case of competitive interactions, by transferring the corresponding σ−τ plot into a plot representing the evolution of the intensities of the two individual odours smelled in a series of mixtures in which the B component is progressively more enriched, beginning by a solution containing pure A. This is represented in Figure 5.

The plot in the left is the σ−τ representation of a mixture displaying a strong compromise effect, similar in magnitude to that reported by Thomas-Danguin and Chastrette [50] for the mixture ethyl salicylate (minty, wintergreen) with heptyl acetate (pear odour). Compromise implies that the addition of odour B causes a decrease on the overall odour intensity of the mixture, whose odour intensity is well below that of A, the most intense component. The plot on the right illustrates how this compromise translates into the odour properties of the mixture, assuming that the pattern of dominance is the one given in Figure 4b (average masking). The plot in Figure 5b clear shows that the addition of little amounts of B, 0.1–0.25 odour units, to A has as single effect the strong decrease of the intensity of the odour A, while the B odour is barely perceived.

These destructive effects can take place at levels close or even below threshold. An example is the effects of the mixture diacetyl, acetoin, acetic acid and ɣ-butyrolactone on a fruity mixture [51]. Destructive interactions are also reported in works dealing with molecules such as volatile phenols including guaiacol (burnt-like aroma), *o*-cresol (tar-like aroma) or ethylphenols (anima/leather/barnyard), 2-iso-butyl-3-methoxypyrazine (earthy/green pepper), 2,4,6-trichloroanisole (mouldy), acetic acid (vinegar) or higher alcohols (spirit-like) [52,53,54,55] that are able to decrease the overall intensity of fruity or woody wine-like models at peri- and sub-threshold levels. Based on these studies it can be hypothesised that molecules imparting odours with negative valence are more likely to be involved in the destructive interactions caused on odour objects with positive valence (e.g., fruity, sweet or woody). However, this hypothesis should be confirmed with further experiments.

Destructive interactions require a strong degree of perceptual interaction. It can be suggested that the loss of intensity is caused by a strong disruption of the signal at peripheral level or by a significant “deconfiguration” of the odour concept at central levels. In the first case, the suppressor would act as antagonist for some of the olfactory receptors activated by the A odour, and in the latter, the suppressor may add a feature that somehow disfigures the odour object represented. Both possibilities involving interactions at either central or peripheral level are not mutually exclusive [56]. 

### 4.3. Cooperative Interactions

Cooperative interactions are those in which the mixture of odours has clear additive (not synergic) effects on the odour intensity without relevant changes in odour quality. These effects can be found at supra-threshold levels but are particularly frequent and characteristic at threshold levels. 

#### 4.3.1. Between Odorants at Threshold Levels

The existence of a strong additivity at threshold levels was first documented in 1963 by Guadagni et al. [57], who observed that mixtures of similar or even different odorants at concentrations below their thresholds could in fact be detected, suggesting that at those levels OAVs are perfectly additive. This fact has been repeatedly confirmed in many different mixtures. Although many studies carried out on the cooperative effects of sub-threshold odours are generally limited to the question of detection, reports from different authors suggest that the mixtures of subthreshold levels of odours convey a common and general sensory note, such as sweet, fresh, harsh or green, which was a common feature of the different odour components of the mixture. This aspect can be particularly relevant in complex mixtures with many components sharing some similarity in aroma. In these cases, the concerted action of a number of subthreshold odorants can induce a perceivable and different odour note. For instance, the concerted action of many different components with flowery-sweet notes formed by the hydrolysis of glycosidic aroma precursors, such as vanillin-derivatives, some volatile phenols, lactones, cinnamates and terpenols on floral-sweet notes of wines has been observed [58]. Similar cooperative effects have been also observed between subthreshold levels of volatile phenols [59], or between sub-threshold levels of geraniol, linalool and citronellol [60] and linalool, geraniol and 3-sulfanyl-4-methylpentan-1-ol in beers [61]. A cooperative effect among unsaturated C6 aldehydes at peri-threshold levels has been also observed in the formation of green and grassy notes of Oolong tea infusions [62]. The additive effects of peri-threshold levels of terpenols on chrysantemun essential oils has been also recently demonstrated [63].

In the past there was some confusion about whether the cooperation between sub-threshold odorants was simply additivity or whether it could qualify as truly synergic. In this respect, it should be noted that the hyper-additivity of OAVs is not enough for determining the existence of synergy. As discussed elsewhere [30], this requires working specifically with the detectability functions of the mixtures. This means that some of the claims of synergism may be simply additive effects and, conversely, that some of the effects tentatively classified as additive could in fact represent synergy. There are few well documented cases demonstrating the existence of synergy between odorants at threshold levels, such as those of Miyazawa et al. [64] about peri-threshold mixture interactions between maple lactone and carboxylic acids and the recent works from Chen et al. [65] about perithreshold levels of aldehydes in Cheddar cheese. 

#### 4.3.2. With or between Odorants at Supra-Threshold Levels 

Cooperative interactions between odorants at threshold levels and odours at suprathreshold levels or between odorants at suprathreshold levels have been also described and seem to be limited to similar odours. The best studied case is that of the ethyl esters present in fruits, wine and other alcoholic beverages. These compounds are responsible for the backbone of the fruity perception in these products. In this regard, it has been demonstrated that sub-threshold levels of some fruity esters such as ethyl 2-hydroxy-4-methylpentanoate (ethyl leucate) [66], ethyl propanoate, ethyl-3-hydroxybutanoate, butyl acetate and isobutyl acetate [67] have significant effects on the intensity and threshold of an ester reconstitution. Mao et al. [68] have also demonstrated that the addition of subthreshold levels of ethyl 2-methylbutanoate, ethyl octanoate, ethyl butanoate and ethyl hexanoate to apple juice solutions decreased the threshold and increased the intensity of the odour of the mixture. The relevant quantitative role played by sub- and peri-threshold ethyl esters has been also demonstrated for the intensity of the ethyl ester vector of red wines [69] and of Langjiu liquor [70]. 

In the recent study from de-la-Fuente-Blanco, Saenz-Navajas, Valentin and Ferreira [69] the qualitative and quantitative effects of the interactions between ethyl esters in wine aroma are analysed. The authors were able to replace the 14 ethyl esters naturally occurring in wine, of which only six were at levels above threshold, by just one of them without any discernible change in the odour quality of an oaky wine. The single requisite was that the monocomponent ester vector had the same intensity as the vector containing the 14 esters. In the case of a wine with a simpler aroma, three different esters had to be retained for a perfect reproduction. In any case, what this demonstrates is something that most flavourists have known for years: that minor components displaying odours similar to those of suprathreshold odorants play qualitatively minor roles. Their qualitative effect is integrated within the most intense similar odour, the integration being the stronger the more complex the odour mixture is.

On the contrary, these minor components play an outstanding relevance in the intensity of the odour of the mixtures. As the original 14-ester vector was progressively simplified by removing minor esters, the concentration in OAV terms had to be much increased to maintain the intensity. In fact, the removal of the eight esters at subthreshold levels, which in OAV terms was equivalent to removing less than 1% of the odour power in the mixture, required to increase the concentration of the remaining esters by more than 50% to maintain isointensity. Overall, to keep the intensity of the original 14-component ester vector, aroma concentration in OAV terms had to increase by nearly a factor 5 in the final monocomponent ester vector. 

**Figure 6:**
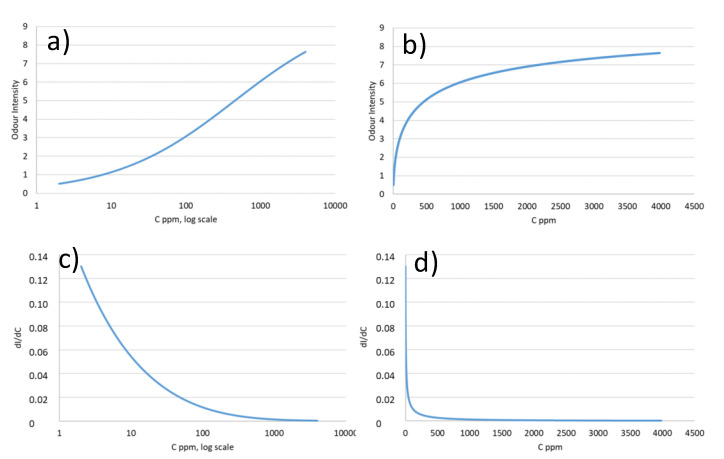
A typical psychophysical plot obtained by the function proposed by Chastrette et al. [71], with $I_{max}\;$= 10, $C_{ip}\;$ = 450 mg/L (concentration at the inflection point), *n* = 0.54 and threshold = 2 mg/L and its first derivative. (**a**,**b**) represent the concentration vs. odour intensity in standard logarithmic and lineal concentration scales, respectively; and (**c**,**d**) the concentration vs. the first derivative of the functions in standard logarithmic and lineal concentration scales, respectively.

Before defining this as a case of synergy, we should take a look into the mathematical characteristics of the psychophysical functions that relate odour intensity with concentration as discussed in reference [69]. As we are used to see these functions in log scale (Figure 6a), in which they look like the logistic function, we forget that in linear scale the functions are power functions (Figure 6b). The first derivative of these functions (Figure 6c,d) is a monotonous ever decreasing function, which displays huge differences in sensitivity between the threshold area and the high-supra threshold area. Simple maths reveal that the intensity added by a concentration unit at threshold equals to that of 43 concentration units at I = 5 for a Stevens coefficient *n* = 0.54. This explains the disproportionate relevance of odorants present at low OAVs in all the previous reports and represents a major challenge for the correct interpretation of the aroma intensities of complex vectors integrating odorants at sub and peri-threshold levels.

### 4.4. Creative Interactions

Creative interactions occur when a series of aroma vectors, present at specific intensity ratios in an odour mixture, trigger a “configurational recognition process” ending in the recognition of an odour object with odour properties different from those of the isolated odorants constituting the mixture. It has been shown that slight changes in the intensity ratios of the odours cause losses of typicality, increasing the difficulty in odour recognition [36,72,73]. Examples of creative interaction in wine can be found in the work from San-Juan, Ferreira, Cacho and Escudero [55], who showed that for a given content in fruity esters, fresh fruit notes were associated to low-intermediate levels of norisoprenoids (β-damascenone and β-ionone) because at higher levels of these compounds, the quality changed to dried fruit notes. In the presence of methional (cooked potato descriptor in isolation) the odour changed to raisin. It has been also observed that the most appreciated “blackcurrant” odour note in red wine can be induced by different mixtures which seem to have in common the ester fruity vector and different combinations/concentrations of other odorants. For instance, 3-mercaptohexyl acetate [74], dimethyl sulphide [75] or even woody extractable aroma compounds at certain ratios [76].

To our understanding, there is a subtype of creative interactions behind the many more or less spectacular aroma enhancing effects noted with relatively frequency in aroma formulation and in natural flavour chemistry. Even if there are not many well documented cases, flavourists come often across with mixtures in which tiny amounts of an odorant cause a disproportionally huge effect on the odour intensity and eventually also on its quality. This is certainly the case of the mixture of heptyl acetate and ethyl salicylate, studied by Moskowitz et al. [77] and by Thomas-Danguin and Chastrette [50], which is one of the few binary mixtures of odorants at suprathreshold levels in which clear hyperadditive or synergic effects regarding intensity have been described. In such a mixture, the addition of 0.33 odour intensity units of ethyl salicylate on 1 odour intensity unit of heptyl acetate, brings the odour intensity of the mixture to 1.84. The magnitude of this synergy becomes evident if this effect is translated into concentrations. Assuming that both odorants have similar psychophysical plots with the same thresholds and a Stevens exponent of 0.4, the amount of salicylate added would be just 6.4% of that of heptyl acetate and produce an increase in odour intensity equivalent to an increase in concentration of heptyl acetate of 460%. Attending to the original report, the addition made the mixture smells more to pear than heptyl acetate itself, whose descriptor is pear-like. 

In our experience, the clearest and serendipitous example came nearly 20 years ago in the reconstitution of a neutral white wine [74]. Before the addition of just 1 ppt of 4-methyl-4-mercaptopentanone, the smell of the reconstitution was very far from being wine-like. It lacked freshness, was too sweet, too synthetic and flat. However, with the mercaptan, the mixture became wine-like and its intensity boosted. Other similar cases are the effects of 3-mercaptohexyl acetate on wine fruitiness and freshness [74,78], of DMS on berry-fruit notes [75], the synergic effects of (E)-2-hexenyl hexanoate, (Z)-3-hexenol, or indole and 4-hexanolide in jasmine tea infusions [79], or the effects of subthreshold levels of acetic acid on maple syrup [80].

In this subtype of creative interaction, the addition of the additional components does not create a completely new odour, but perfects or completes an already existing one. In this respect, it should be considered that the odours of natural products, with which our odour concepts are formed via familiarisation, are blends of different odorants playing different roles in the odour concept. In a number of cases, a few odorants communicate to the odour of the product an essential part of its quality, so that the odours of these odorants are defined using the name of the product. For instance, the odour of ethyl 2-methylbutyrate is defined as apple-like or strawberry-like, and the odour of vanillin is vanilla. However, other secondary odorants, such as fatty acids in the case of apple [55] or guaiacol or anisaldehyde and anisyl alcohol in the case of natural vanilla [81,82] communicate complementary but relevant attributes, so that their presence makes the odour more real or typical, boosting its intensity. As the scientific literature demonstrates, any clue helping the subjects to identify an odour assigning it to the corresponding memorised odour concept produces an increase on intensity, pleasantness and familiarity [83]. 

## 5. A Systematic Approach Based on Perceptual Interactions to Interpret the Role of Odorants in the Formation of Complex Odours. The Case of Wine Aroma

**Figure 7:**
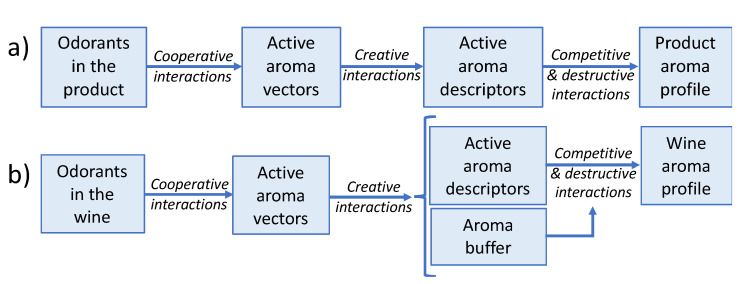
Schemas showing the use of perceptual interactions for interpreting the role of compositional odorants in the final sensory descriptors of any product (**a**) or specifically of wine (**b**).

The approaches for a general case and specifically for wine are summarised in the schemas given in Figure 7. The obvious first step is to have a list with the odorants present in the original product, including a measurement of their intensity. Then, similar odorants in this list will be grouped into homogeneous aroma vectors to produce the list of the active aroma vectors present in the product. Over this list, there is a quite complicated task, which is to identify the different types of creative interactions within the list of aroma vectors to deduce the list of active aroma descriptors.

Creative interactions include both the enhancement effects in which secondary aroma vectors integrate into a main aroma vector, boosting the intensity but not changing much the quality, and those others in which genuine new aroma nuances emerge. The list of active aroma descriptors, translates then into the product sensory profile by applying the rules of dominance (competitive interactions), and if there are in the odour mixture odour suppressors, applying also the extra odour suppression effects linked to destructive interactions.

In the particular case of wine, there is a specific element, not yet perfectly studied, which is the aroma buffer [76] or the wine aroma signature [84]. This element is formed by ethanol and by the 27 major fermentative aroma compounds present in all fermented beverages at levels close and above threshold. This complex mixture determines not only liquid-gas partition coefficients, since ethanol increases the solubility of most aroma compounds, but also influences mass transfer phenomena and strongly influences sensory properties. First, because it provides a continuous and strong aroma background displaying a “vinous” aroma of its own against which the different odour objects have to be recognised. In this sense, it plays the role of “olfactory white” in analogies to visual and auditory “whites” as stated by Weiss et al. [85]. Second, because it has notable suppressing effects towards most odours [54], which implies that it will be particularly active in destructive interactions.

At present, research has advanced to identify most qualitative elements of the systematic approach. The odorants of wine, their integration via cooperative interactions into active aroma vectors, and the odour descriptors which these active aroma vectors can elicit within the wine aroma buffer, are given in Table 1. For the next years, it is expected to have advances in the different quantitative aspects of the approach to make it fully operative.

## 6. Conclusions

Odour interactions in complex mixtures can be classified into four major categories: competitive, cooperative, destructive and creative. These categories are based in and are consistent with classical psychophysical laws and include also the most important odour blending effects by incorporating concepts related to image processing. Competitive and cooperative interactions are soft modes of odour interactions with a limited concourse of configurational or top-down processing, while destructive and creative are hard modes of interaction in which top-down processing is essential.

These interactions can be applied to complex odorant systems following the order: cooperative creative, destructive and competitive; to transform odorant intensities into aroma profiles. While the major concepts seem to be well established, there are yet a number of difficulties related to our poor handling of odour intensities and the yet limited number of studies about different aspects of creative interactions, including studies at both cognitive and receptor levels.

## Figures and Tables

**Table 1:** Odorant composition of the updated active vectors and active aroma descriptors of wine.

Active Odour/Descriptor	Active Aroma Vector	Odorants within the Vector	Sensory Descriptors
Alcoholic/solvent	*Ethanol*	Ethanol	Solvent, sweet, harsh
	*Fusel alcohols*	Isobutyl alcohol and isoamyl alcohol, β-phenylethanol *	Harsh, spirit, solvent
	*Ethyl acetate*	Ethyl acetate	Glue
Lactic/acid	*Diacetyl*	Diacetyl, 2,3-pentanedione *	Buttery, milky, yogurt,
	*Fatty acids*	Butyric acid, hexanoic acid, octanoic acid and decanoic acid	Soapy, cheese
	*Branched acids*	Isobutyric acid, isovaleric acid and 2-methylbutyric acid	Sweaty, cheese
Reduced	*Hydrogen sulphide and mercaptans*	Hydrogen sulphide, methanethiol, ethanethiol	Rotten egg, cooked cabbage
Yeasty, oxidized	*Acetaldehyde*	Acetaldehyde	Green apple, oxidised
	*Isoaldehydes*	Isobutanal, 2-methylbutanal and 3-methylbutanal	Yeasty, malty
	*Methional*	Methional	Potato, overripe oxidised
Flowery	*Phenylacetaldehyde*	Phenylacetaldehyde	Honey, oxidised
	*Phenylethyl acetate*	Phenylethyl acetate	Floral, sweet, rose
	*Cinnamates*	Ethyl cinnamate, ethyl dihydrocinnamate	Sweet, balsamic
	*β-ionone*	β-ionone, α-ionone *	Violets, berry
	*Terpenols*	Linalool, geraniol, nerol *, citronellol *, α-terpineol *	Jasmine, orange blossom, muscat
	*Cis-rose oxide*	Cis-rose oxide	Rose, litchi
Fruity	*Ethyl esters*	Ethyl butyrate, ethyl hexanoate, ethyl octanoate *, ethyl decanoate *, ethyl propanoate *, ethyl isobutyrate, ethyl 2-methylbutyrate, ethyl 3-methylbutyrate, ethyl 4-methylpentanoate, ethyl hydroxybutyrate *, ethyl 2-methylpentanoate *, ethyl 3-methylpentanoate *, ethyl cyclohexanoate *, 2-hydroxy-4-methylpentanoate*	Fruity, apple, strawberry, blackberry, anise
	*β-damascenone*	β-damascenone	Baked apple, dry plum
	*Furaneol*	Furaneol, homofuraneol, maltol	Strawberry, sugary
	*Isoamyl acetate*	Isoamyl acetate, isobutyl acetate *	Banana
	*γ-lactones*	γ-octalactone, γ-nonalactone, γ-decalactone, γ-undecalactone and γ-dodecalactone	Peach
Citric/green	*3-mercaptohexyl acetate*	3-mercaptohexyl acetate	Passion fruit
	*3-mercaptohexanol*	3-mercaptohexanol	Grapefruit
	*4-methyl-4-mercaptopentan-2-one*	4-methyl-4-mercaptopentan-2-one	Box tree, fresh
	*Piperitone*	Piperitone	Minty, fresh
Vegetal	*Alkylpyrazines*	3-Isopropyl-2-methoxypyrazine, 3-Isobutyl-2-methoxypyrazine and 3-sec-Butyl-2-methoxypyrazine	Green pepper, green, earthy
Spice/woody	*Vanillas*	vanillin *, ethyl vanillate *, methyl vanillate *, acetovanillone *	Vanilla, nutmeg
	*Whiskylactone*	Z- whiskylactone and E-whiskylactone *	Oaky, coconut
	*Volatile phenols*	Eugenol, isoeugenol, guaiacol, 4-methylguaiacol *, vanillinthiol	Clove, smoky
	*Sotolon*	Sotolon	Liquorice, curry
	*Rotundone*	Rotundone	Black or white pepper
	*Dimethyl sulphide*	Dimethyl sulphide	Black truffle, black olive
	*1,1,6-Trimethyl-1,2-dihydronaphthalene*	1,1,6-Trimethyl-1,2-dihydronaphthalene	Kerosene
Empyreumatic	*Furfuryl thiol*	Furfuryl thiol	Coffee, roasted
	*Benzyl mercaptan*	Benzyl mercaptan	Charred, smoke, toasted

* Aroma compounds within the vectors that most probably are not sensory relevant.
